# Allostasis as a Conceptual Framework Linking Bipolar Disorder and Addiction

**DOI:** 10.3389/fpsyt.2014.00173

**Published:** 2014-12-03

**Authors:** Mauro Pettorruso, Luisa De Risio, Marco Di Nicola, Giovanni Martinotti, Gianluigi Conte, Luigi Janiri

**Affiliations:** ^1^Department of Neuroscience, Institute of Psychiatry and Clinical Psychology, Catholic University of Sacred Heart, Rome, Italy; ^2^Department of Neuroscience and Imaging, Institute of Psychiatry, “G. d’Annunzio” University of Chieti-Pescara, Chieti, Italy

**Keywords:** bipolar disorders, addiction vulnerability, allostasis and allostatic load, comorbidity, hedonic tone and anhedonia, dopaminergic system, reward system, CRF/HPA axis and stress system

## Abstract

Bipolar disorders (BDs) and addictions constitute reciprocal risk factors and are best considered under a unitary perspective. The concepts of allostasis and allostatic load (AL) may contribute to the understanding of the complex relationships between BD and addictive behaviors. Allostasis entails the safeguarding of reward function stability by recruitment of changes in the reward and stress system neurocircuitry and it may help to elucidate neurobiological underpinnings of vulnerability to addiction in BD patients. Conceptualizing BD as an illness involving the cumulative build-up of allostatic states, we hypothesize a progressive dysregulation of reward circuits clinically expressed as negative affective states (i.e., anhedonia). Such negative affective states may render BD patients more vulnerable to drug addiction, fostering a very rapid transition from occasional drug use to addiction, through mechanisms of negative reinforcement. The resulting addictive behavior-related ALs, in turn, may contribute to illness progression. This framework could have a heuristic value to enhance research on pathophysiology and treatment of BD and addiction comorbidity.

## Introduction

Bipolar disorder (BD) is a severe, often chronic condition with lifetime prevalence rates of up to 6.5% for bipolar spectrum disorders in the general population (1). BD patients frequently report co-occurring substance-use disorders (SUDs) and behavioral addictions (1–5). The rates of alcohol and other SUDs are significantly higher in subjects with BD than in the general population (1, 6). The co-occurrence of BD and addiction has important clinical implications (3, 7). Bipolar patients with comorbid conditions present with a more severe course of illness (8), characterized by an overall worse clinical picture (9), poorer treatment outcome (10–12), higher suicidality (13), and mortality (14).

Several studies have aimed to identify the endophenotypical features predisposing to the development of addiction in the general population, as well as in the context of BD. These studies focused on genetic vulnerability, impulsive traits, and decision-making impairment (15–19).

The aim of this paper is to present the possible contribution of the concept of allostasis as a framework linking BD and addiction. We hypothesize that the concepts of allostasis and allostatic load (AL) may contribute to the understanding of the complex relationships between BD and addictive behaviors (20–22). Allostasis entails the safeguarding of reward function stability by recruitment of changes in the reward and stress system neurocircuitry (21) and it may help to elucidate neurobiological underpinnings of vulnerability to addiction in BD patients.

## Methods

Computerized database, i.e., PubMed, Psycinfo, Cochrane Library were searched using the following terms: “allostasis,” “AL,” “reward,” “hedonic tone,” “stress system” cross-referenced with “BD,” “addiction,” and “SUDs.” The results of this search are presented in this article, and examined in light of a unifying hypothesis with a potential heuristic value to inform and provide direction to future research in this intriguing area.

## Relevance of Allostasis in BD and Addiction Field

### Bipolar disorders

Bipolar disorders is a complex and multifactorial disease, with genetic and environmental factors contributing to its clinical expression (23). BD can also be conceptualized as an illness involving the cumulative build-up of allostatic states, whereas AL progressively increases as stressors and mood episodes occur over time (24). Indeed, it has been postulated that mood episodes function as allostatic states, generating a load that is responsible for illness progression commonly seen in BD (25, 26). AL may contribute to a better understanding of BD, particularly of inter-episodic phenomena such as vulnerability to stress, cognitive symptoms (26), and higher physical comorbidity rates (24). BD patients present with alterations in major mediators of AL. They exhibit for instance, persistent dysregulation of the hypothalamic-pituitary-adrenocortical (HPA) axis, circadian rhythm disturbances, altered immunity as well as pro-inflammatory and oxidative stress states [please refer to the review Kapczinski et al. (24)]. Neurotrophic factors play an important role in maintaining a physiological brain function. They have been shown to be modulated by environmental events in various psychopathological conditions (27), and their role has been confirmed also in pathophysiology and staging of BD (28–31).

These alterations are greater during the acute stages of the disease, but remain sub-threshold even during remission (24). When mediators of allostasis – essential for brain functioning and protection – are driven by mechanisms of homeostatic dysregulation, they act in excess and damage brain tissue (32, 33), which is particularly vulnerable to the harmful effects of the AL [i.e., oxidative stress (34)]. Impairment in the stress response has been acknowledged as a core feature of BD clinical expression, as well as having a central role in the concept of AL (23). Although the exact mechanisms, by which stress exerts its effect on the brain, remain largely unknown, the HPA axis is one of the main stress response systems activated with the objective to maintain stress adaptation for as long as it is necessary (23). The HPA axis is clearly altered in mood disorders, as well as in BD (35–38). Glucocorticoids play an important role in the process whereby the mediators of allostasis interact with neurotransmitter systems and brain peptides resulting in neuroplastic alterations in the hippocampus, amygdala, and prefrontal cortex (39, 40). The role of stress in triggering mood episodes is well established, particularly in the early stages of illness (41, 42). It has been hypothesized that early life stress could affect the endocrine system, producing a stable reprograming of HPA axis (43), leading to an impairment in brain area involved in emotional processing (44). Alterations in emotional processing involving amygdala circuitry and are related to BD symptoms in several ways. Evidence from amygdala-dependent tasks points to a dysregulation of amygdala-related neurocircuitry in BD patients (45). These alterations render BD patients more prone to trigger AL (23), through a greater stress vulnerability.

### Addiction

Drug addiction can be conceptualized as a stress-surfeit disorder (46). It is characterized by the occurrence of an allostatic state in the brain reward system, reflected in a chronic deviation of reward thresholds (46–48). An allostatic state reflects a new balance, a state of chronic deviation of the regulatory system from its normal (homeostatic) operating level to a pathological (allostatic) operating level (47). From a drug addiction perspective, repeated compromised activity in the dopaminergic system and sustained activation of the corticotropin-releasing factor (CRF) system may lead to an AL that contributes significantly to the transition from occasional drug use to drug addiction (49, 50). This model may be applied to pathological gambling as well (51). The transition from occasional controlled drug use to loss of control is endorsed by the emergence of negative affective states, resulting from the abovementioned allostatic dysregulations (i.e., the AL), with a shift from impulsivity to compulsivity and from positive reinforcement to negative reinforcement (49, 52).

Addiction implies dysregulation of the brain reward system (48, 53). Several studies highlighted that negative affective states are a result of the alteration of neurobiological elements central to reward and stress systems (50, 54, 55), in brain areas such as the ventral striatum and the extended amygdala (56, 57). In addition to the reduction of dopaminergic and opioidergic functioning, dysregulation of reward is also mediated by the activation of brain stress systems (i.e., CRF), in the areas of the extended amygdala (57). Stress system alterations have been observed in both the acute and chronic phases of addiction, and seem to play a role in determining reward dysregulation (48, 54). Acute withdrawal raises the threshold for reward, leads to an increase in dysphoric symptoms as well as an increase of CRF levels in the amygdala (49, 58). These changes result from sensitization of the brain stress system in response to the phenomena of abstinence, and persist for a long period of time following cessation of drug intake [protracted withdrawal (59)]. Protracted withdrawal symptoms are related to the compulsivity characterizing addictive disorders, and are factors involved in determining relapse. In addition to CRF, other mediators (norepinephrine, dynorphin, and neuropeptide Y) have been investigated and found to play a role in the transition from impulsivity to compulsivity (58, 60). As a whole, these elements constitute the brain stress system of the extended amygdala, a counter-adaptive system that interacts with the reward system and determine its reduced function (48).

## Neurobiological Issues of BD-SUD Comorbidity

A complete review of neurobiological features in BD-SUD comorbidity is beyond the purpose of this paper. Familial and illness course characteristics of BD and addictive disorders, as well as shared underlying mechanisms suggest potentially important genetic overlap (19, 61, 62). Preliminary findings hint at the existence of a shared genetic vulnerability for BD and SUDs (15). Johnson et al. (63) found convergent genome-wide association results for BD and SUDs. Products of one group of these genes are likely to play substantial roles in the initial and/or plasticity-related “wiring” of the brain (63). A second group of genes is the family of clock genes, implicated in the regulation of behavioral and physiological periodicity (19). Recently, a significant genetic overlap between candidate genes for both alcoholism and BD was found (64, 65), by using the d-box binding protein knockout mouse, a stress-reactive animal model developed consistently with allostasis and stress-surfeit theory of addiction (46).

To date, no studies have specifically investigated neuroimaging correlates in comorbid BD–SUD patients. Several studies describe putative mechanisms involved in BD vulnerability to addiction. Structural imaging studies in BD patients found volume reductions in prefrontal cortex [PFC (66)], which is involved in encoding incentive information (67). During Iowa gambling task (IGT), BD patients showed abnormalities in the dorsal and ventral PFC, while lateral temporal and polar regions displayed increased activation (68). Jogia et al. (69) confirmed these observations and also reported a greater activation in the anterior cingulate cortex of BD patients performing the IGT and in the insula during the n-back working memory task. Reduced functioning of the dopamine transporter (DAT) has been linked to BD (70–72). Animal models may provide insight into the role of the dopaminergic system in risk-taking behavior. Mice with reduced DAT functioning exhibit a behavioral profile consistent with manic patients and increased risk-taking behavior during a mouse version of the IGT (70). Evidence from these animal model studies and translational human research in BD subjects (73, 74), allows us to hypothesize that system-related change involving functioning of the dopamine system play a role in impulsive choice, risk-taking behavior, and reward, thus help guiding future studies in BD–SUD subjects.

## Allostatic Dysregulation of Reward Might Underpin Bipolar Vulnerability to Addiction

Dopaminergic mechanisms are likely to play a key role in the understanding of the pathophysiology of BD and the clinical phenomena of mania and depression have previously been conceptualized in terms of an increase or a decrease in dopaminergic function, respectively (75, 76). Also, converging lines of evidence suggest that dopamine is a key neurotransmitter mediating hedonic allostasis in drug and behavioral addictions (49, 77). From a neurobiological perspective, a central dopaminergic dysfunction has been widely proposed as a neurobiological correlate of anhedonia (78). Different studies suggest anhedonia as a key symptom in addictive disorders, both as part of a withdrawal syndrome and as a relevant factor involved in relapses (51, 59, 79). In addition to dopamine, other neurotransmitters are believed to encode the hedonic experience [endogenous opioids, serotonin (80)], while long-lasting alterations involving cue-induced craving and relapse are thought to result from neuroplastic changes in glutamatergic circuitry (81–83).

Several studies provide support for reward dysregulation accounts in BD (16, 18, 45, 69, 84–95) (Table 1), characterizing neural dynamics underlying inter-temporal reward processing (90). Possibly emotional dysregulation present in BD is related to hypersensitivity to reward-relevant stimuli (93). Impulsive and unsafe decision-making in BD is linked to decreased sensitivity to emotional contexts involving rewards or punishments, possibly reflecting altered appraisal of prospective gains and losses associated with certain behaviors (89). It has been proposed that anhedonia could be mediated by a change in reward sensitivity (78), which has different behavioral consequences involving either stress-related and dopaminergic processes (96). In BD, sustained allostatic states and the consequent cumulative brain damage resulting from increased AL may play a part in the occurrence of negative affective states (i.e., anhedonia) that persist even during periods of remission (84). Counter-adaptive processes, such as opponent process that are part of the normal homeostatic limitation of reward function (55) fail to return within the normal homeostatic range and are hypothesized to repeatedly drive the allostatic state [decreased dopamine and opioid peptide function, increased CRF activity (49)]. This allostatic state is hypothesized to be reflected in a chronic deviation of reward set point that is fueled, not only by dysregulation of reward circuits *per se* but also by recruitment of brain and hormonal stress responses.

**Table 1:** **Reward-system alterations and vulnerability to addiction in euthymic bipolar patients**.

Aim	Methods	Sample	Results	Comments	Reference
Trait-related decision-making impairment	IGT, sensitivity-to-punishment index	167 BD (45 mania, 32 depressed, 90 euthymic), 150 HC	Manic, depressed, and euthymic BPs selected significantly more cards from the risky decks than HC. BD preferred decks that yielded infrequent penalties over those yielding frequent penalties.	BD have a trait-related impairment in decision-making that does not vary across illness phase, predicted by high depressive scores	(16)
Decision-making deficits; temporal discounting of reward	Delay discounting task	22 BD, 21 SZ, 30 HC	BD and SZ groups discounted delayed rewards more steeply than did the healthy group (even after controlling for current substance use). Working memory or intelligence scores negatively correlated with discounting rate.	BD patients value smaller, immediate rewards more than larger, delayed rewards	(18)
Neural mechanisms related to motivation	fMRI, probabilistic reversal learning task	19 BD, 19 HC, 22 relatives, 22 HC	Increased activation in response to reward and reward reversal contingencies in the left medial orbitofrontal cortex in BD. Activation of the amygdala in response to reward reversal was increased.	Increased activity of OFC and amygdala, related to heightened sensitivity to reward and deficient prediction error signal	(45)
Functional brain abnormalities during reward and working memory processing	fMRI, IGT, n-back task	36 BD, 37 HC	BD showed inefficient engagement within the ventral frontopolar prefrontal cortex with segregation along the medial–lateral dimension for reward and working memory processing, respectively. Greater activation in the anterior cingulate cortex during the IGT and in the insula during the n-back task.	Over-activation in regions involved in emotional arousal is present even in tasks that do not typically engage emotional systems	(69)
Hedonic capacity	SHAPS, SANS-An, VAS-HC	107 BD, 86 MDD, 106 HC	SHAPS, SANS-An, and VAS scores significantly higher in affective disorder patients. 20.5% of BDs showed significant reduction in hedonic capacity	Reduced hedonic capacity persists irrespective of mood state	(84)
Relationship between SUD and overweight-obesity	Data from CCHS, BMI	36,984 individuals	Overweight/obese bipolar individuals had a lower rate of SUD than the normal weight sample (13 vs. 21%). BD + SUD had a lower rate of overweight/obesity when compared with BD non-SUD (39 vs. 54%)	Comorbid addictive disorders may compete for the same brain reward systems	(85)
Neural correlates of reward and decision-making	IGT, RDMUR, ERP-assessed RDGT	13 BD, 12 ADHD, 25 HC	BD group showed a pattern of enhanced ‘learning by feedback’ and ‘sensitivity to reward magnitude’ regardless of valence. This ERP pattern was associated with mood and inhibitory control. Reduced responses of the cingulate cortex to the valence and magnitude of rewards in BD.	Altered decision-making process in BD with the involvement of cingulate cortex	(86)
Impulsivity	BIS-11, stop signal task, delayed reward task, continuous performance task	108 BD1 (1-year FU), 48 HC	At baseline (manic/mixed state), BD demonstrated significant deficits on all three tasks. Performance on the three behavioral tasks normalized upon switching to depression or developing euthymia. Elevated BIS-11 scores persist across phases of illness.	Impulsivity has both affective-state dependent and trait components in BD.	(87)
Dysfunctional reward processing	Probabilistic reward task	18 BD, 25 HC	BD showed a reduced and delayed acquisition of response bias toward the more frequently rewarded stimulus	Dysfunctional reward learning in situations requiring integration of reinforcement information in BD	(88)
Risky decision-making (rewards vs punishments)	Risky decision-making task	20 BD-2, DF, 20 HC	The BD participants overestimated the number of bad outcomes arising out of positively framed dilemmas. Risky choice in BD is associated with reduced sensitivity to emotional contexts that highlight rewards or punishments.	In BD, altered valuations of prospective gains and losses associated with behavioral options.	(89)
Neural correlates of hypersensitivity to immediate reward	(1) Two choice impulsivity paradigm (2) Delay discounting task, EEG	1) 32 subjects 2) 32 subjects	(1) The hypomania-prone group made significantly more immediate choices than the control group. (2) The hypomania-prone group evidenced greater differentiation between delayed and immediate outcomes in early attention-sensitive (N1) and later reward-sensitive (feedback-related negativity) components.	Provide support for reward dysregulation accounts of BD, characterizing neural dynamics underlying inter-temporal reward processing	(90)
Substance sensitivity and sensation seeking	SCID-I, SCI-SUBS	57 BD1-SUD, 47 BD1, 35 SUD, 50 HC	BD + SUD and SUD have higher scores on self-medication, substance sensitivity and sensation seeking. No differences in reasons for substance use between BD + SUD and SUD (improving mood; relieving tension; alleviating boredom; achieving/maintaining euphoria; increasing energy).	In BD patients, substance sensitivity and sensation seeking traits are possible factors associated with SUD development	(91)
Reward sensitivity and positive affect	RPA; RRI; BQL-BD	90 BD1, 72 HC	The majority of BD-1 reported avoiding at least one rewarding activity as a means of preventing mania. Lower quality of life related to dampening positive emotions.	People with BD-1 report avoiding rewarding activities and dampening positive emotion	(92)
Neural correlates of hypersensitivity to reward	fMRI, anticipation and outcome reward task	21 BD1, 20 HC	BD displayed greater ventral striatal and right-sided OFC (BA 11) activity during anticipation, but not outcome, of monetary reward. BD also displayed elevated left-lateral OFC (BA 47) activity during reward anticipation	Elevated ventral striatal and OFC activity during reward anticipation as a mechanism underlying predisposition to hypo/mania in response to reward-relevant cues.	(93)
Sensitivity to positive and negative feedback	Learning task (positive/negative feedback)	23 BD1, 19 MD, 19 HC	The quality of the last affective episode was the only significant predictor. BD1 patients who last experienced a manic episode learned well from positive but not negative feedback, whereas BD1 patients who last experienced a depressive episode showed the opposite pattern	Differences in response to positive and negative consequences carrying over into the euthymic state are related to the polarity of the preceding episode	(94)
Motivational aspects of decision-making in relation to reward and punishment	IGT	28 BD (14 acute and 14 remitted) 25 HC	Acute BD were characterized by the tendency to make erratic choices. Low choice consistency improved the prediction of acute BD beyond that provided by cognitive functioning and self-report measures of personality and temperament.	Low choice consistency in BD patients	(95)

*BD, bipolar disorder; SZ, schizophrenia; HC, healthy controls; SUD, substance-use disorder; SCID-I, structured clinical interview for DSM-IV axis I disorders; SCI-SUBS, structured clinical interview for the spectrum of substance use; DF, drug-free; SHAPS, Snaith–Hamilton pleasure scale; SANS-An, scale for the assessment of negative symptoms, subscale for anhedonia/asociality; VAS-HC, visual analog scale for hedonic capacity; BIS-11, Barratt Impulsiveness Scale; RPA, responses to positive affect measure; BQL-BD, brief quality of life in bipolar disorder scale; RRI, reward responses inventory; IGT, Iowa gambling task; RDMUR, task of rational decision-making under risk; RDGT, rapid-decision gambling task; ERP, event-related potentials; fMRI, functional magnetic resonance imaging; EEG, electroencephalography; CCHS, Canadian Community Health Survey-Mental Health and Well-Being; OFC, orbitofrontal cortex; BA, Brodmann area; FU, follow-up*.

Altered functioning of the HPA axis may hold clues to the nature of the motivational changes accompanying addiction and vulnerability to addiction (97). Pre-existing alterations in frontal–limbic interactions with the HPA may reflect addiction-proneness, as shown in studies of offspring of alcohol- and drug-abusing parents (98). Alterations in the CRF/HPA axis may exert effects on the corticostriatal-limbic motivational, learning, and adaptation systems that include mesolimbic dopamine, glutamate, and gamma-amino-butyric acid (GABA) pathways (97), representing the underlying pathophysiology associated with stress-related risk of addiction.

The effects of these allostatic changes in the mesocorticolimbic brain system and in CRF/HPA axis contribute to the underlying pathophysiology associated with stress-related risk of addiction in BD (99). In BD patients, we hypothesize that the hedonic response to an acute drug administration occurs on a pre-existing allostatic dysregulation of the dopamine and CRF system. BD-related allostatic alterations in reward and stress systems thereby constitute vulnerability factors to the development of addiction in subjects exposed to occasional drug use. The failure to self-regulate these systems, determined by the collective contribution of endogenous factors linked to BD and of exogenous substances, results in an AL leading to a facilitated transition to drug addiction.

**Figure 1:**
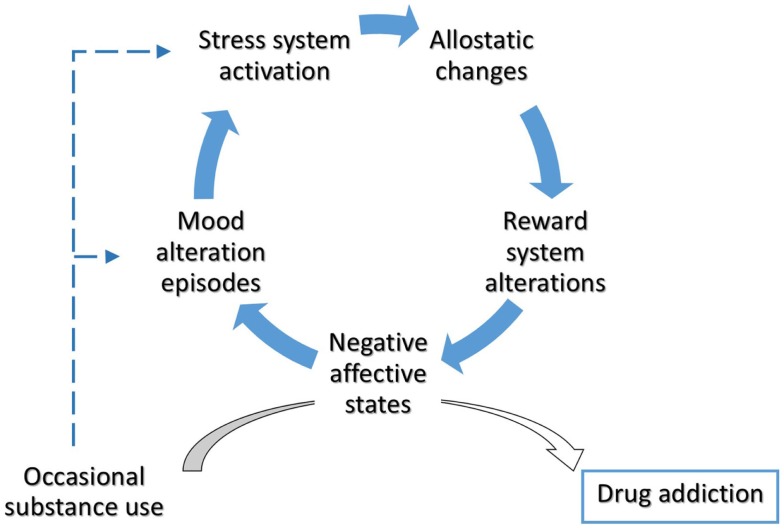
**Allostatic alterations in bipolar disorder and vulnerability to addiction**. Throughout the involvement of enduring alterations in stress- and reward-system, BD patients could experience a rapid transition from occasional drug use to drug addiction. The occurrence of negative affective states mediate the switch from impulsivity to compulsivity in bipolar patients. Cumulative effects of mood episodes and substance use on stress system have been hypothesized.

Dysphoria triggers drug intake, accompanied by an intense activity of the dopaminergic system and followed by a compensatory decrease in the dopaminergic system and increase in the CRF system to re-establish the allostatic set point. Such negative affective states may render BD patients more vulnerable to drug addiction, favoring a very rapid transition from occasional, recreational drug use to compulsive, pathological, drug dependence. The resulting addictive behavior-related ALs, in turn, may contribute to illness progression (Figure 1).

## Clinical Implications and Future Perspectives

Converging data from addiction and BD studies suggest that these disorders involve similar allostatic processes, and allostasis can contribute to unify these disorders under a unitary perspective. In this context, the concepts of allostasis and AL provide both a pathophysiological model for the understanding of BD-addiction comorbidity and a new perspective for the development of novel therapeutic strategies for the treatment of comorbid patients (100, 101).

Allostatic alterations in brain reward system could render BD patients more vulnerable to drug addiction, favoring a very rapid transition from occasional, recreational drug use to compulsive, pathological, and drug dependence. This framework allows us to explain the high comorbidity rate between these disorders (2), as well as its relevance in early-onset patients (8, 102). Furthermore, it enables us to identify the factors of vulnerability to addiction in inter-episode periods as well (i.e., sub-threshold reward-system dysfunctions) (84). A more accurate monitoring of comorbidity-risk (103), coupled with the inclusion of specific tools for the assessment of hedonic tone, may contribute to early intervention on addiction-vulnerability factors and to initiate primary prophylaxis for substance misuse in youth suffering from BD with high-risk for addiction (104–106).

Currently, accruing evidence suggests that mood alteration episodes increase the risk of substance use (107, 108). Patients with dual disorders are more likely to use substances to self-regulate perceived internal factors (109, 110). SUD comorbidity in BD patients was preceded by greater manic symptoms in the previous period (104), as well as the persistence of depressive symptoms was associated with higher craving and increased risk to develop substance dependence (104, 108). Moreover, in gambling disorder (GD) patients depressive symptoms predicted gambling urges and duration (111). Allostasis framework enables us to extend the self-medication theory (112) beyond the established clinical domains, increasing the understanding of the interactions between BD symptoms and substance use. For instance, euthymic bipolar patients are more likely to experience cognitive impairment (deficits in measures of executive functions, verbal learning, immediate and delayed verbal memory, abstraction, sustained attention) (113). Cannabis abuse seems to positively affect cognitive function in a BD sample (114), and it may represent an attempt to counterbalance these alterations, even though causing an increased risk of rapid cycling and an earlier onset of manic episodes (114, 115).

Practitioners should be particularly vigilant in monitoring for substance misuse early after the onset of mood disorders, as well as they should be aware of personality traits related to the risk of addiction, in particular antisocial and schizotypal personality disorder (11, 116). The existence of additional risk factors [i.e., ADHD (117)] for the development of a BD-SUD comorbidity is controversial (105, 118). Combined with a specific role of traumatic stress as independent vulnerability-factor (99, 119), these elements contribute to the build-up of a cumulative AL. Clinicians can therefore incorporate specific therapy approaches for dual disorders (120–122) to target adherence weaknesses (123) and to enhance the effects of existing treatments.

Given the notion that exposure to stress or drugs leads to enduring changes in gene expression or activation of transcription factors, determining long-term neuroadaptation of brain functions, a promising field of future research could involve the detection of valuable markers of AL (124). In fact, markers of AL could contribute to prevention strategy (105, 116, 125); moreover, they could improve clinical monitoring and prognostic assessment of comorbid patients.

The clinical management of BD-SUD subjects requires a careful distinction between mood and withdrawal/intoxication symptoms (126, 127). Neuroimaging studies indicate that brain regions involved in mood regulation lie in close proximity to regions involved in motivation and craving (128). The complex interplay between addiction and BD domains, mediated by the involvement of similar neurobiological systems, requires further studies to better delineate how BD and SUD operate as reciprocal risk factors (105, 129). Recently, it has been proposed to focus on some clinical domain by using strategies aimed to treat both disorders simultaneously (101, 130). Besides reducing the recurrence of affective episodes, and exerting neuroprotective, mood stabilizers have been recently shown to have anti-anhedonic properties (131–134) with potential utility in the treatment of comorbid conditions (135–141). In addition, glutamatergic agents have been demonstrated to be effective in the treatment of both mood (142) and addictive disorders (82, 143); furthermore, they have been recently proposed as a valuable therapeutic option in the treatment of comorbid patients (139).

Future studies aimed at assessing brain AL in patients with BD and addiction comorbidity may help to shed light on the complex interactions underlying neurobiological vulnerability to these disorders and to improve their treatment options. Early effective treatment strategies specifically devised for comorbid patients (104, 125) could prevent, or possibly reverse, some of the neurobiological abnormalities and indicators of AL, thus potentially leading to numerous benefits for these patients.

## Conflict of Interest Statement

The authors declare that the research was conducted in the absence of any commercial or financial relationships that could be construed as a potential conflict of interest.

## Abbreviations

AL, allostatic load; BD, bipolar disorder; CRF, corticotropin-releasing factor; GD, gambling disorder; HPA, hypothalamic-pituitary-adrenocortical (axis); SUD, substance-use disorder.
